# VAP1 promotes cardiac fibrosis by enabling PDGFR signaling in myofibroblasts

**DOI:** 10.1038/s12276-026-01690-7

**Published:** 2026-04-20

**Authors:** Shan Huang, Qianwen Zhao, Tinghui Shao, Chenghao Zhu, Yujia Xue, Naxia Chen, Yue Zhang, Huihui Xu, Ming Kong, Rui Wang

**Affiliations:** 1 Hainan Provincial Key Laboratory for Tropical Cardiovascular Diseases Research and Key Laboratory of Emergency and Trauma of Ministry of Education, Institute of Cardiovascular Research, Department of Cardiology, The First Affiliated Hospital, Hainan Medical University, Haikou, China; 2 Department of Infectious Diseases, Nanjing Drum Tower Hospital Affiliated with Nanjing University Medical School, Nanjing, China; 3 Department of Pathology, Nanjing Drum Tower Hospital Affiliated with Nanjing University Medical School, Nanjing, China; 4 State Key Laboratory of Natural Medicines, Department of Pharmacology, China Pharmaceutical University, Nanjing, China; 5 Department of Cardiovascular Surgery, Nanjing First Hospital, Nanjing Medical University, Nanjing, China; 6 Key Laboratory of Targeted Intervention of Cardiovascular Disease and Collaborative Innovation Center for Cardiovascular Translational Medicine, Nanjing Medical University, Nanjing, China

**Keywords:** Heart failure, Experimental models of disease

## Abstract

Excessive fibrogenesis is associated with adverse cardiac remodeling and heart failure. Myofibroblast, primarily derived resident fibroblast, is the effector cell type in cardiac fibrosis. The mechanism whereby fibroblast–myofibroblast transition is driven remains incompletely understood. In the present study, we investigated the role and targetability of vascular adhesion protein 1 (VAP1) in cardiac fibrosis. Transcriptomic screening identified VAP1 as a direct target for megakaryocytic leukemia 1 (MKL1), a master regulator of tissue fibrosis. VAP1 silencing in primary cardiac fibroblasts down-regulated expression of myofibroblast markers and weakened cell proliferation/migration/contraction when exposed to transforming growth factor-β, whereas VAP1 over-expression exerted the opposite effects. Importantly, VAP1 deletion in quiescent fibroblasts or activated fibroblasts (myofibroblasts), achieved through the *Col1a2*-Cre driver and the *Postn*-Cre driver, respectively, dampened cardiac fibrosis and rescued heart function in mice subjected to the transverse aortic constriction procedure. Data obtained from multi-omics techniques indicated that VAP1 influenced fibroblast–myofibroblast transition by directly interacting with platelet-derived growth factor receptor-beta to enable signal transduction. Finally, small-molecule VAP1 inhibitors attenuated cardiac fibrosis and improved heart function in mice. In conclusion, our data support a role for VAP1 in driving fibroblast activation and cardiac fibrosis. Therefore, targeting VAP1 can be considered as a reasonable approach for the intervention of heart failure.

## Inhibiting VAP1 Alleviates Fibrosis and Enhances Heart Recovery

Heart failure (HF) is a serious health issue worldwide, similar in severity to cancer. Despite medical advances, its prevalence is rising owing to aging populations and increasing penetrance of obesity and diabetes. Researchers are trying to understand the molecular basis underlying HF to develop better treatments. A key factor in HF is cardiac fibrosis, which involves myofibroblasts. This study explores the role of a protein called vascular adhesion protein 1 (VAP1) in cardiac fibrosis. Researchers used mice with specific genetic modifications to study the role of VAP1 in fibroblasts and myofibroblasts. They found that removing VAP1 from fibroblasts/myofibroblasts reduced heart scarring. They also discovered that VAP1 could activate platelet-derived growth factor receptor-beta signaling, which may contribute to fibrosis. The study concludes that targeting VAP1 could be a promising strategy to reduce cardiac fibrosis and to improve heart function in patients with HF.

## Introduction

Heart failure (HF), defined by a multisociety consensus statement as a clinical syndrome with symptoms and/or signs caused by a structural and/or functional cardiac abnormality and corroborated by elevated natriuretic peptide levels and/or objective evidence of pulmonary orsystemic congestion^1^, is a major health burden worldwide with comparable mortality rates as cancers. Despite the advances in diagnostic, preventive, and interventional techniques, HF prevalence has continued to increase in the past decade and is projected to increase further in the next decade largely owing to an increasingly aging population and the epidemics of obesity and diabetes globally^2^. These findings highlight an urgent need to delineate the molecular mechanisms underlying HF for the development of stratified and personalized treatment strategies. Pathological ventricular remodeling, characterized by diffuse myocardial fibrosis, represents a key contributing factor to HF^3^. Although spatiotemporally controlled fibrogenic response is integral to the host defense mechanism following injury safeguarding myocardial homeostasis and preserving heart function, aberrant myocardial fibrosis invariably disrupts anatomical and functional integrity of the myocardium leading up to HF^4^.

Regardless of etiology, myofibroblasts are the primary effector cell type in myocardial fibrosis^5^. As a cell lineage specialized in producing extracellular matrix (ECM) proteins to remodel the ventricles, myofibroblasts are transient in nature only detectable in the course of cardiac fibrosis^6^. Decades of research have led to a consensus among those working in the field that resident cardiac fibroblasts are the predominant progenitors from which ECM-producing myofibroblasts are derived in cardiac fibrosis^7^. Fibroblast–myofibroblast transition is regulated by a complex network of signaling cascades initiated by a host of fibromodulatory growth factors, cytokines, and hormones^8^. Platelet-derived growth factor (PDGF)–PDGF receptor (PDGFR) signaling represents one of the most prominent pro-fibrogenic pathways that contribute to cardiac fibrosis. It has been previously shown that mice harboring myocardial-specific over-expression of various isoforms of PDGF (that is, PDGF-A, PDGF-B, and PDGF-D) develop spontaneous cardiac fibrosis^9,10^. On the contrary, blockade of the PDGF/PDGFR signaling with small-molecule antagonists or neutralizing antibodies is associated with dampened cardiac fibrosis^11,12^.

Vascular adhesion protein 1 (VAP1), also known as amine oxidase containing 3 (AOC3), was initially characterized by Jalkanen and co-workers as an endothelial surface glycoprotein that supports leukocyte rolling by catalyzing amino group deamination and covalent bond formation^13,14^. Weston et al.^15^ have previously demonstrated that VAP1 deletion in mice attenuates CCl_4_-induced liver fibrosis. Consistently, Marttila-Ichihara et al.^16^ have later reported that the same VAP1 knockout (KO) mice developed less extensive pulmonary fibrosis than wild-type littermates when administered with bleomycin. Of interest, a recent report by Hsia et al.^17^ proposes that VAP1 might be a bona fide myofibroblast marker. However, because both the studies of Weston et al. and Marttila-Ichihara et al. have used global VAP1 KO mice as opposed to lineage conditional KO mice, it is not immediately clear whether the observed fibrosis phenotype was a result of defective fibroblast activation/myofibroblast maturation or secondary to compromised leukocyte trafficking as documented in both studies. In the present study, we sought to assign a fibroblast–myofibroblast autonomous role for VAP1 in cardiac fibrosis. Our data suggest that fibroblast–myofibroblast-specific deletion of VAP1 is sufficient to mitigate cardiac fibrosis. VAP1 contributes to fibroblast activation likely by enabling PDGFR signaling.

## Methods

### Animals

All animal experiments were reviewed and approved by the Ethics Committee on Humane Treatment of Laboratory Animals of Nanjing Medical University (approval no.: IACUC-2109008-1). All animal procedures conform to the guidelines from Directive 2010/63/EU of the European Parliament on the protection of animals used for scientific purposes or the NIH guidelines for the Care and Use of Laboratory Animals. Unless specified, the animals were kept under constant environmental conditions with 12 h light–dark cycles and ad libitum access to food and water. Myofibroblast-specific MKL1 KO mice (*MKL1*^ΔMF^) were generated by crossing the *MKL1*^f/f^ mice to the *Postn*-Cre^ERT2^ mice^18^ and have been described previously^19^. Myofibroblast-specific MKL1 knock-in mice (*MKL1*^MFTg^) were generated by crossing the *MKL1*^Rosa/+^ mice to the *Postn*-Cre^ERT2^ mice and have been described previously^20^. Vap1^f/f^ mice were generated by floxing the first exon of the murine *Vap1* gene. The *Col1a2*-Cre^ERT2^ mice^21^ have been previously described. To induce Cre expression in the *Col1a2*-Cre^ERT2^ mice, tamoxifen (T2859, Sigma, St Louis, MO, USA) was injected peritoneally (50 mg/kg) for 7 consecutive days followed by a washing phase of 7 days as previously described^21,22^. To induce Cre expression in the *Postn*-Cre^ERT2^ mice, tamoxifen was injected peritoneally (50 mg/kg) for 5 consecutive days followed by maintaining the mice on a TAX-containing diet (130855, Inotiv, Lafayette, IN, USA), as previously described^18,23^.

For the transverse aortic constriction (TAC) procedure, 8–10-week male mice (25–27 g) were anesthetized using 5 mg/ml ketamine plus 2 mg/ml xylazine. The hearts were exposed through left thoracotomy in the third intercostal space. A 1.0 mm wire was placed alongside the transverse aorta, which was tied to the wire between the first and second branches of the aortic arch. The wire was quickly removed, leaving the aortic arch constricted to the diameter of the wire. The mice were sacrificed 6 weeks after the surgery. In certain experiments, the mice were injected with a VAP1 inhibitor (PXS-4728A, HY-112726, MedChemExpress, Monmouth Junction, NJ, USA) thrice a week at a dose of 10 mg/kg. Cardiac functions were evaluated by echocardiography (GE Vivid 7 equipped with a 14-MHz phase array linear transducer, S12, allowing a 150 maximal sweep rate). M-mode imaging was applied at the level of the papillary muscles to assess left ventricular (LV) dimensions. Representative echo images can found in the Supplementary material (Supplementary Fig. 6). All data were obtained under steady-state conditions with minimal respiratory influence, and values were averaged over at least three consecutive cardiac cycles. Offline analysis was performed using VEVO Lab software (version 5.6.1). The animals were euthanized to carbon dioxide inhalation to obtain their samples.

### Cell culture and treatments

Human embryonic kidney cells HEK293 (CRL-1573, ATCC, Manassas, VA, USA) were maintained in DMEM (11995065, Thermo Fisher, Waltham, MA, USA) supplemented with 10% fetal bovine serum (A5209501, Thermo Fisher). Primary human cardiac fibroblasts (ventricular cardiac fibroblasts isolated from normal, adult heart tissue) were purchased (CC-2904, Lonza, Basel, Switzerland) and maintained in FBM^TM^ Basal Medium (CC-3131) and FGM^TM^-3 SingleQuot Supplements (CC-4525). Typically, the primary cells were used between passages 3 and 6. Primary mouse cardiac fibroblasts were isolated, as previously described^20^. Briefly, the ventricles were dissected from 8-week-old C57B/6j mice and minced into small pieces with a microscopic scissor and then digested with type II collagenase (17101015, Thermo Fisher) for 60 min in a cell culture incubator. The digested cells were passed through a 100 μm cell strainer (352360, Corning, Charlotte, NC, USA) and pelleted by centrifugation. The pellet was re-suspended in complete DMEM culture media and placed in a cell culture incubator for 4 h, after which time the media (along with the floating cells) were carefully aspirated. The attached cells were maintained in complete DMEM culture media and used for experiments between passage 3 and passage 6. The cells were serum-starved overnight before recombinant TGF-β (5 μg/ml, 7666-MB-005, R&D, Minneapolis, MN, USA) or angiotensin II (Ang II) (1 μM, A9525, Sigma) was added in serum-free media.

### DNA plasmids, transient transfection, and reporter assay

Small interfering RNAs (siRNAs) were obtained from Dharmacon (J-010143-05, Beijing, China). Lipofectamine 3000 (L3000015, Thermo Fisher, for DNA vectors)and Lipofectamine RNAiMax (13778150, Thermo Fisher, for siRNAs) were used for transient transfections. The *Vap1* promoter luciferase construct was generated by amplifying genomic DNA spanning the proximal promoter and the first exon of the *Vap1* gene (−2000/+100) and ligating into a pGL4-basic vector (E6651, Promega, Madison, WI, USA). FLAG-tagged VAP1 and HA-tagged PDGFR-beta (PDGFRβ) vectors were generated by standard molecular cloning technique. QuickChange II kit (200519, Agilent, Santa Clara, CA, USA) was used for mutagenesis. For transient transfection, the cells were seeded at a density of 2 × 10^5^ cell per well (for 6-well culture dish) or 1 × 10^5^ cells per well (for 12-well culture dish) the day before transfection. DNA plasmids or siRNAs were mixed with the transfection reagents (for DNA plasmids, Lipofectamine 3000 was used at a ratio of 3:1; for siRNAs, Lipofectamine RNAiMax was used at a ratio of 2:1) and added to the cells. The cells were harvested 24–48 h after transfection. Reporter assay was performed with a luciferase assay system, as per vendor's instructions (E1500, Promega).

### Protein extraction, immunoprecipitation, and western blotting

Whole-cell/tissue lysates were obtained by re-suspending pellets in RIPA buffer (50 mM Tris pH 7.4, 150 mM NaCl, 1% Triton X-100), with freshly added protease inhibitors (A32955, Thermo Fisher), as previously described^24^. For immunoprecipitation (IP), protein lysates were pre-cleared with Protein A/G-Plus Agarose beads (SC-2003, Santa Cruz Biotechnology, Santa Cruz, CA, USA) for 30 min on a 360 ^o^C rotator. About 5 μg of each of the appropriate antibodies was added to the pre-cleared proteins and the mixture was incubated on a 360 ^o^C rotator at 4 ^o^C overnight. Protein complex formed thereafter was absorbed by Protein A/G-Plus Agarose beads for additional 3 h. Precipitated immune complex was released by boiling with 1× SDS electrophoresis sample buffer. Equal amounts of proteins (~25 μg) were separated by 10% mini SDS–PAGE and transferred to nitrocellulose membranes (1620112, Bio-Rad, Hercules, CA, USA). The membranes were blocked with 5% milk powder in TBST (Tris-buffered saline with Tween-20) buffer at room temperature for 1 h and then hybridized at room temperature to the following commercially available antibodies: anti-VAP1 (14365-1, Proteintech, Wuhan, China), anti-FLAG (F3165, Sigma), anti-HA (26183, Thermo Fisher), anti-PDGFRβ (ab69506, Abcam, Cambridge, UK), anti-phospho-PDGFRβ (ab218534, Abcam), anti-JNK (9252, Cell Signaling Tech, Danvers, MA, USA), anti-phospho-JNK (4668, Cell Signaling Tech), anti-ERK (4695, Cell Signaling Tech), anti-phospho-ERK (4370, Cell Signaling Tech), anti-p38 (9212, Cell Signaling Tech), anti-phospho-p38 (4511, Cell Signaling Tech), and anti-β-actin (A2228, Sigma) for 3 h. After three washes with TBST buffer, the membranes were incubated with appropriate secondary antibodies conjugated to horseradish peroxidase for another 1 h at room temperature. Protein blots were visualized using ECL reagent (A38554, Thermo Fisher) on a Kodak image station (NEN, Boston, MA, USA). For densitometrical quantification, densities of target proteins were normalized to those of loading controls. Data are expressed as relative protein levels compared with the control group, which is arbitrarily set as 1. All experiments were repeated at least three times.

### Chromatin immunoprecipitation

Chromatin IP assays were performed essentially, as described before^25^. In brief, chromatin in control and treated cells was cross-linked with 1% formaldehyde. Cells were incubated in lysis buffer (150 mM NaCl, 25 mM Tris pH 7.5, 1% Triton X-100, 0.1% SDS, 0.5% deoxycholate) supplemented with protease inhibitor tablet. DNA was fragmented into ~200 bp pieces using a Branson 250 sonicator (Emerson, St Louis, MO, USA). Aliquots of lysates containing 200 μg of protein were used for each IP reaction with 5 μg of anti-MKL1 (21166-1, Proteintech) or pre-immune IgG. Precipitated genomic DNA was amplified by real-time PCR. About 10% of the starting material was included as the input. Data are expressed as relative enrichment (%) compared with input.

### EdU incorporation assay

5-Ethynyl-2ʹ-deoxyuridine (EdU) incorporation assay was performed in triplicate wells with a commercially available kit (C10337, Thermo Fisher). Briefly, the EdU solution was diluted with the culture media and added to the cells for an incubation period of 2 h at 37 ^o^C. After several washes with 1× phosphate-buffered saline, the cells were then fixed with 4% formaldehyde and stained with Alexa Fluor™ 488. The nucleus was counterstained with 4′,6-diamidino-2-phenylindole. The images were visualized by fluorescence microscopy and analyzed with Image-Pro Plus (Media Cybernetics). For each well, six different fields were randomly chosen and the positively stained cells were counted and divided by the number of total cells. The average of the six fields for each well was calculated. The averages of each group were then normalized to the averages of the control group. The data are expressed as relative EdU staining compared with the control group arbitrarily set as 1.

### Boyden chamber migration assay

The cells were trypsinized and seeded into Boyden chambers (Polyester track-etched, 8-μm pores, 24-well format; 354597, Becton Dickinson, Franklin Lakes, NJ, USA) in serum-free DMEM medium. Complete culture medium containing 10% fetal bovine serum was added to the lower chamber. The cells migrating from the upper chamber were fixed with 4% paraformaldehyde, stained with 0.1% crystal violet, and counted under a microscope. Cell numbers from five random fields were counted in each well. The data are expressed as relative cell migration compared with the control group arbitrarily set as 1.

### Collagen contraction assay

The cells were trypsinized, mixed with four times the volume of Collagen Gel Working Solution (354236, Corning) and incubated for 1 h at 37 °C. After collagen polymerization, 1.0 ml of culture medium was added atop. The collagen gel size change was measured 24 h later and quantified with Image-Pro Plus. Data are expressed as relative contraction normalized to the control group arbitrarily set as 1.

### Picrosirius Red staining

Picrosirius Red (PSR) staining was performed using a commercially available kit (ab150681, Abcam). Following dewaxing and rehydration, paraffin sections were immersed in PSR staining solution and incubated for 1 h at room temperature under light-protected conditions. Subsequently, the sections were rinsed twice with 0.5% acetic acid solution to remove nonspecific background staining. After conventional dehydration through a graded ethanol series and clearing, the sections were mounted with neutral gum. Following staining, images were captured using an optical microscope (DMI8, Leica, Wetzlar, Germany) at 10× magnification, and whole-slide scans were obtained using a stereo microscope (M60, Leica) for macroscopic observation.

### Masson’s trichrome staining

Masson’s trichrome staining was performed using a commercial kit (ab150686, Abcam). Following dewaxing and rehydration, paraffin sections were stained sequentially as follows: acid fuchsin solution for 4 min, rinsed with 0.1% acetic acid for 1 min; differentiated with phosphomolybdate solution for 1–2 min, rinsed again with 0.1% acetic acid for 1 min; and counterstained with aniline blue solution for 4 min, followed by a final rinse with 0.1% acetic acid for 1 min. Sections were then dehydrated through a graded ethanol series to remove residual stain, cleared, and mounted with neutral gum. Following staining, images were captured using an optical microscope (DMI8, Leica) at 10× magnification, and whole-slide scans were obtained using a stereo microscope (M60, Leica) for macroscopic observation.

### Hydroxyproline quantification

Tissue hydroxyproline levels were quantified with a commercially available kit (ab222941, Abcam), as per vendor's recommendation. Briefly, heart tissues (~10 mg) were homogenized with the BeadBlaster microtube homogenizer (D2400, LABRepCo, Horsham, PA, USA). The homogenates were then mixed with NaOH (10N) at 120 °C for 1 h followed by neutralization with HCl (10 N). The samples were centrifuged at 10,000*g* for 5 min. The supernatants were collected and incubated with the Oxidation Reagent Mix in a 96-well microplate at room temperature for 20 min. Developer solution was added and incubated with the samples at 65 °C for 30 min followed by absorbance measurement at an optical density of 560 nm on a SpectraMax ABS microplate reader (Molecular Devices, San Jose, CA, USA).

### RNA-sequencing and data analysis

Transcriptome sequencing was conducted on total RNA isolated from primary murine cardiac fibroblasts under different treatment conditions, with three biological replicates per group. Total RNA was extracted using TRIzol reagent, and RNA integrity was verified by denaturing agarose gel electrophoresis. Sequencing libraries were constructed following standard procedures, including RNA fragmentation and cDNA synthesis, and were subjected to high-throughput sequencing on an Illumina NovaSeq™ 6000 platform. Raw sequencing reads were processed with Trimmomatic (v0.39) to retain only those with a Phred quality score (*Q*-score) >30 for downstream analyses, yielding an average of 40 million reads per sample. High-quality reads were aligned to the murine reference genome (mm39) and gene expression levels were quantified. Genes not expressed in more than 50% of samples were filtered out, and the remaining genes were retained for subsequent analysis. Differential expression analysis was performed using the limma R package (v3.18) on a voom-corrected matrix, with significance thresholds set at |log_2_ fold change | > 0.58 and a *P*-value <0.05. For differentially expressed genes, promoter regions (±2000 bp from the transcription start site) were subjected to de novo motif analysis using HOMER’s findMotifsGenome.pl (v4.11) with the parameters “-size 200 -mask -p 8” and a *P*-value cut-off of <1 × 10^−5^. Gene Ontology (GO) and Kyoto Encyclopedia of Genes and Genomes (KEGG) pathway enrichment analyses were performed using the clusterProfiler R package (v4.0.5) via a hypergeometric test, with all expressed genes serving as the background set; terms with a Benjamini–Hochberg false discovery rate (FDR)-adjusted *P*-value <0.05 were considered statistically significant.

### Mass spectrometry

Primary murine cardiac fibroblasts (2 × 10^6^ cells in a p100 culture dish) were transduced with adenoviral FLAG-tagged VAP1 at an multiplicity of infection (MOI) of 10. Subsequently, cells were lysed and the extracted proteins were subjected to IP using an anti-FLAG antibody (F3165, Sigma). The immunoprecipitated complexes were eluted and denatured with 1× SDS loading buffer, followed by separation via SDS–PAGE. The gel was stained with Coomassie Brilliant Blue, and entire protein lanes were excised and processed for in-gel digestion. Gel pieces were destained, reduced, and alkylated before digestion with 10 ng/μl trypsin in 100 mM NH_4_HCO_3_ at 37 ^o^C for 17 h. The resulting peptides were extracted, and the supernatant was collected after centrifugation at 12,000*g* for 30 min. The peptide extract was then lyophilized, desalted using C18 stage tips, and lyophilized again. The dried peptides were reconstituted in 15 μl of 0.1% formic acid and centrifuged at 12,000*g* for 5 min, and the resulting supernatant was analyzed by liquid chromatography/mass spectrometry (MS) on a Q-Exactive mass spectrometer (Thermo Scientific). Raw MS data were processed using MaxQuant software (version 1.6.5.0) and searched against the appropriate protein sequence database. Protein identification was performed with an FDR threshold set at 1%.

### Bioinformatic analysis of proteomics data

The MS-based proteomics data analyzed in this study were obtained from the PRIDE public repository (Dataset Accession nos PXD026582 and PXD028101). The raw data were uniformly processed using MaxQuant software and searched against the *Mus musculus* reference proteome from UniProtKB. Key analysis parameters included: digestion enzyme (trypsin/P), maximum missed cleavages (2), and PSM and protein FDR threshold (0.01). A standard database of contaminants was included in the analysis. Subsequent analysis was performed in the R environment. The proteinGroups.txt file generated by MaxQuant was imported and filtered to remove reverse sequences, potential contaminants, and proteins only identified by site. Protein LFQ intensities were log_2_-transformed and normalized using the NormalizeBetweenArrays function from the limma package. Differential analysis was conducted using the moderated *t*-test in the limma package, with proteins showing an absolute fold change ≥1.2 and a *P*-value <0.05 defined as differentially expressed proteins.

### Statistical analysis

Statistical analysis was performed with the SPSS package (IBM SPSS v18.0, Chicago, IL, USA). Data are expressed as mean ± SD and sample sizes/replicates are reported in the figure legends. For experiments involving two groups, a Student’s *t*-test was performed. For experiments involving at least three groups, one-way analysis of variance with post hoc Scheffe’s analyses was performed. The assumptions of normality were checked using the Shapiro–Wilk test, and equal variance was checked using Levene’s test; both were satisfied.

## Results

### VAP1 as a novel target downstream of MKL1

**Fig. 1:**
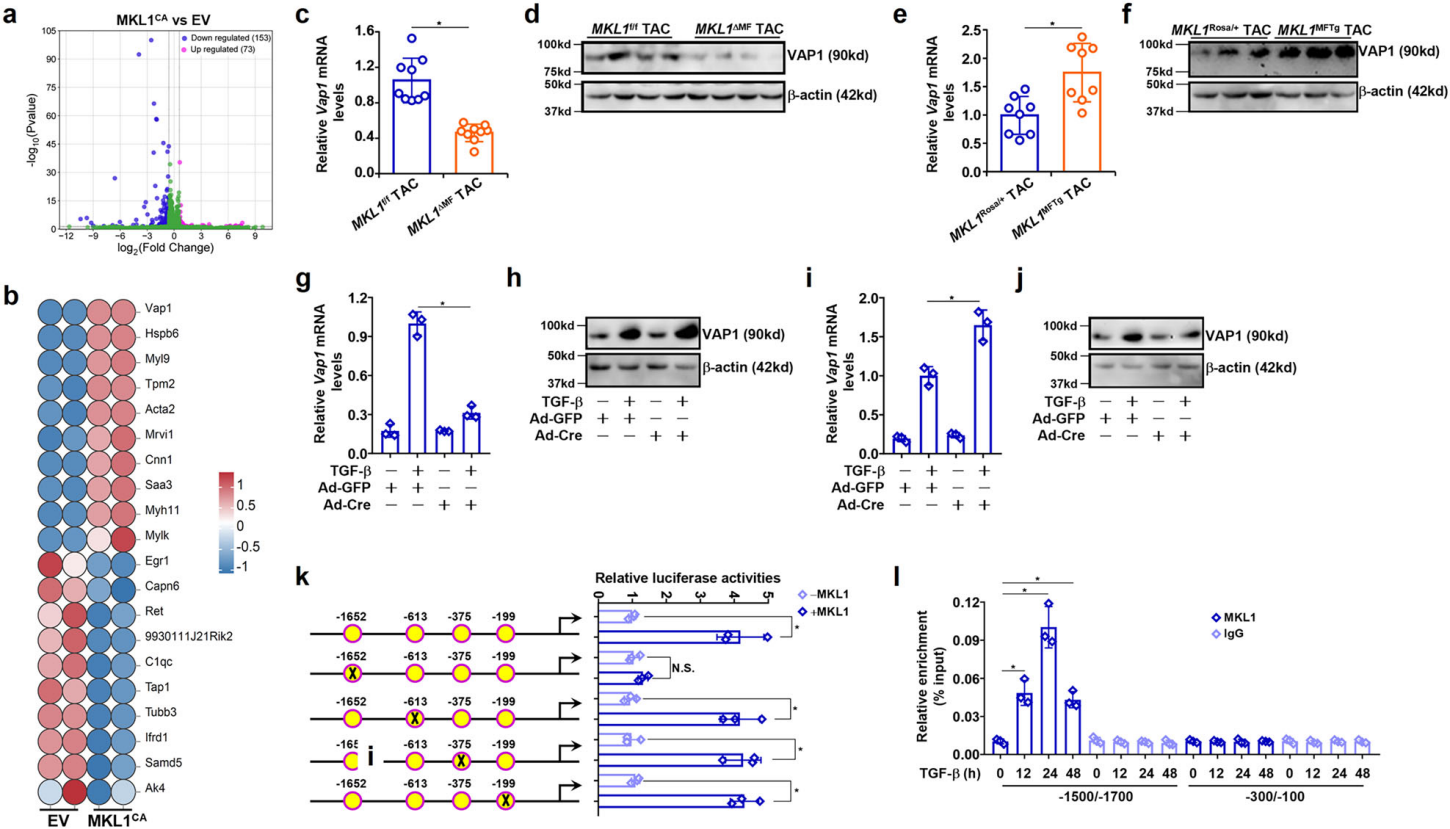
VAP1 is a new MKL1 target. **a**,**b** Primary murine cardiac fibroblasts were transduced with lentiviral MKL1^CA^ or empty vector (EV). RNA-sequencing was performed as described in the Methods section. Volcano plot showing differentially expressed genes in the MKL1^CA^-overexpressing cells versus the control cells (part **a**). Heatmap of differentially expressed genes (part **b**). **c**,**d** MKL1^ΔΜF^ mice and MKL1^f/f^ mice were subjected to the transverse aortic constriction (TAC) procedure and sacrificed 4 weeks after the procedure. Vascular adhesion protein 1 (VAP1) expression was examined by quantitative PCR (qPCR) and western blotting. *n* = 4–8 mice for each group. Data are expressed as mean ± SD. *P < 0.05, Student’s *t*-test. **e**,**f** MKL1^ΜFTg^ mice and MKL1^Rosa/+^ mice were subjected to the TAC procedure and sacrificed 4 weeks after the procedure. VAP1 expression was examined by qPCR and western blotting. n = 4–8 mice for each group. Data are expressed as mean ± SD. *P < 0.05, Student’s *t*-test. **g**,**h** Primary cardiac fibroblasts isolated from the MKL1^f/f^ mice were transduced with adenoviral Cre or GFP followed by treatment with transforming growth factor-beta (TGF-β) (5 ng/ml) for 24 h. VAP1 expression was examined by qPCR and western blotting. *n* = 3 biological replicates. Data are expressed as mean ± SD. *P < 0.05, one-way analysis of variance (ANOVA) with post hoc Scheffe’s test. **i**,**j** Primary cardiac fibroblasts isolated from the MKL1^Rosa/+^ mice were transduced with adenoviral Cre or GFP followed by treatment with TGF-β (5 ng/ml) for 24 h. VAP1 expression was examined by qPCR and western blotting. *n* = 3 biological replicates. Data are expressed as mean ± SD. *P < 0.05, one-way ANOVA with post hoc Scheffe’s test. **k** Wild-type and mutant *Vap1* promoter-luciferase constructs were transfected into HEK293 cells with or without MKL1. Luciferase activities were normalized by GFP fluorescence and protein concentration. *n* = 3 biological replicates. Data are expressed as mean ± SD. *P < 0.05, Student’s *t*-test. **l** Primary murine cardiac fibroblasts were treated with TGF-β (5 ng/ml) and harvested at indicated time points. Chromatin immunoprecipitation assays were performed with anti-MKL1 or IgG. *n* = 3 biological replicates. Data are expressed as mean ± SD. *P <0.05, one-way ANOVA with post hoc Scheffe’s test. NS, not significant.

Previous studies have unequivocally demonstrated that MKL1 deletion in (myo)fibroblasts attenuates, whereas MKL1 over-expression in (myo)fibroblasts enhances cardiac fibrosis in mice^20,26^. Because nuclear translocation serves as a rate-limiting step in MKL1-dependent transcription^27^, a constitutively nucleus-residing MKL1 (MKL1^CA^) was transduced into cardiac fibroblasts followed by RNA-sequencing to identify new MKL1 targets. A total of 226 genes were significantly (1.5×-fold, *p* < 0.05) altered by MKL1^CA^ over-expression with approximately two-thirds being up-regulated (153) and slightly less than one-third being down-regulated (73) (Fig. 1a). Because MKL1 is primarily considered as an activator of transcription^28^, we focussed on genes that were up-regulated by MKL1^CA^ over-expression. VAP1 ranked top (by *Pp*-value) among the genes up-regulated by MKL1^CA^ over-expression (Fig. 1b). When both myofibroblast-specific MKL1 KO mice (*MKL1*^ΔMF^) and wild-type mice (*MKL1*^f/f^) were subjected to the TAC procedure to induce cardiac fibrosis, it was discovered that VAP1 expression was much lower in the *MKL1*^ΔMF^ hearts than in the control hearts (Fig. 1c,d). On the contrary, cardiac VAP1 expression was elevated in the mice harboring myofibroblast-specific MKL1 overexpression (*MKL1*^MFTg^), compared with the control mice (*MKL1*^Rosa/+^) (Fig. 1e,f).

VAP1 was inducible by several different profibrogenic stimuli including TGF-β, Ang II, and endothelin-1 (ET-1) in cardiac fibroblasts (Supplementary Fig. 1). Primary cardiac fibroblasts were isolated from the *MKL1*^f/f^ mice or the *MKL1*^Rosa/+^ mice followed by transduction with adenoviral Cre to deplete or over-express MKL1; MKL1 deletion attenuated (Fig. 1g,h) whereas MKL1 over-expression (Fig. 1i,j) enhanced VAP1 induction by TGF-β. A string of four putative CArG boxes, to which MKL1 can be recruited by serum response factor (SRF)^29^, was identified within 2 kb relative to the transcription start site of the *Vap1* gene; mutation of the most distal CArG box, but not the other three, completely abrogated the responsiveness of the *Vap1* promoter to MKL1 over-expression as indicated by reporter assay (Fig. 1k). Indeed, chromatin immunoprecipitation assay confirmed that TGF-β treatment augmented MKL1 occupancy on the most distal CArG box of the *Vap1* promoter (Fig. 1l). Similar observations were made in heart tissues collected from the mice subjected to the TAC procedure or the sham procedure (Supplementary Fig. 2). Combined, these data identify and validate VAP1 as an MKL1 target in cardiac fibroblasts.

### VAP1 manipulation alters myofibroblast differentiation

**Fig. 2:**
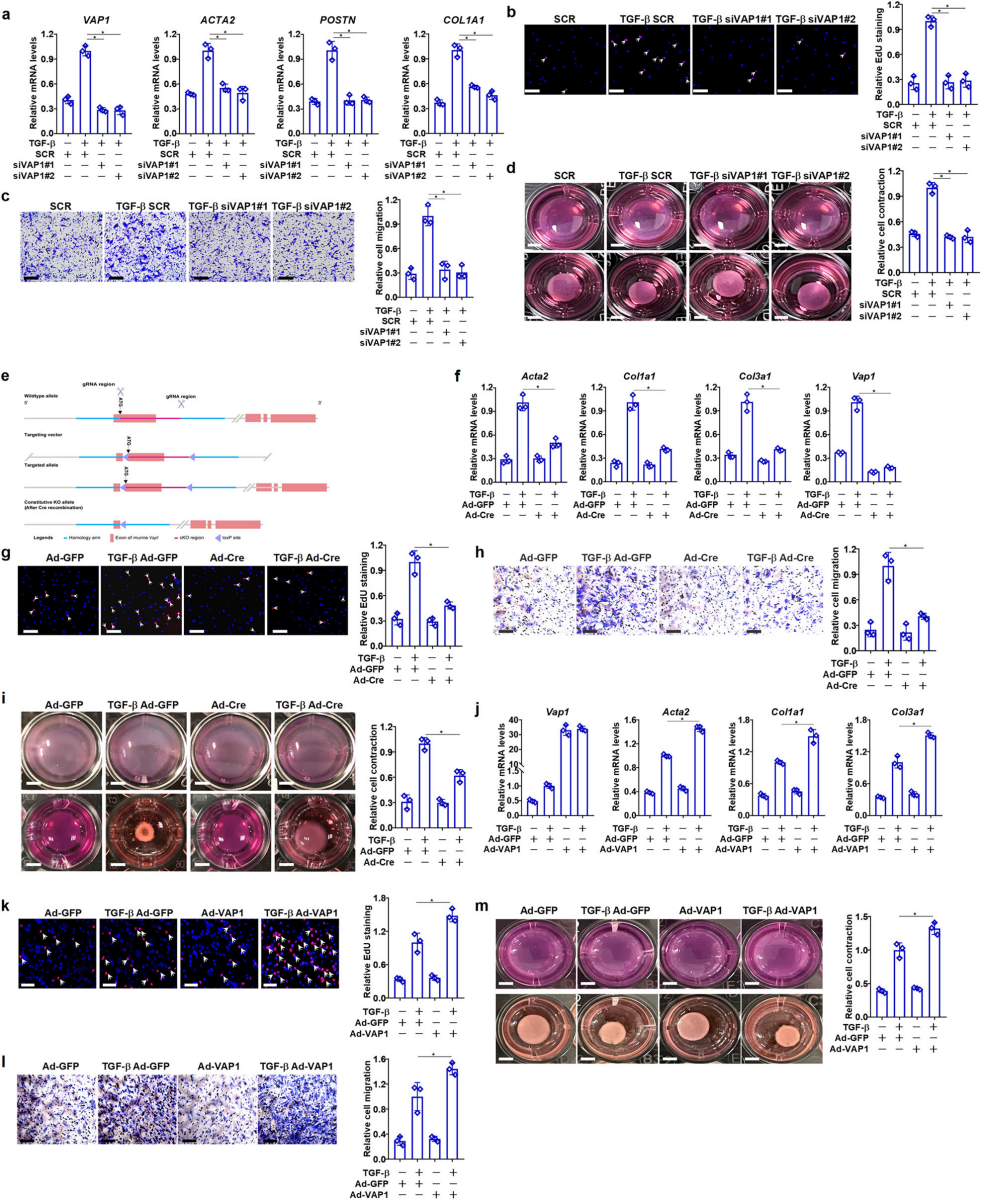
VAP1 manipulation alters myofibroblast differentiation. **a**–**d** Primary human cardiac fibroblasts were transfected with small interfering RNAs targeting VAP1 (siVAP1) or scrambled small interfering RNA (SCR) followed by treatment with transforming growth factor-β (TGF-β) (5 ng/ml) for 24 h. Myofibroblast markers were examined by quantitative PCR (qPCR) (part **a**). 5-Ethynyl-2ʹ-deoxyuridine (EdU) incorporation assay. Scale bar, 50 μm (part **b**). Cell migration was examined by Boyden transwell assay. Scale bar, 50 μm (part **c**). Collagen contraction assay. Scale bar, 1 cm (part **d**). **e** Targeting scheme of the Vap1^f/f^ mice. **f**–**i** Primary cardiac fibroblasts isolated from the Vap1^f/f^ mice were transduced with Ad-Cre or Ad-GFP followed by treatment with TGF-β (5 ng/ml) for 24 h. Myofibroblast markers were examined by qPCR (part **f**). EdU incorporation assay. Scale bar, 50 μm (part **g**). Cell migration was examined by Boyden transwell assay. Scale bar, 50 μm (part **h**). Collagen contraction assay. Scale bar, 1 cm (part **i**). **j**–**m** Primary murine cardiac fibroblasts were transduced with Ad-VAP1 or Ad-GFP followed by treatment with TGF-β (5 ng/ml) for 24 h. Myofibroblast markers were examined by qPCR (part **j**). EdU incorporation assay. Scale bar, 50 μm (part **k**). Boyden transwell assay. Scale bar, 50 μm (part **l**). Collagen contraction assay. Scale bar, 1 cm (part **m**). *n* = 3 biological replicates. Data are expressed as mean ± SD. *P < 0.05, one-way analysis of variance with post hoc Scheffe’s test. gRNA guide RNA, KO knockout, VAP1 vascular adhesion protein 1.

A series of experiments were performed to verify whether VAP1 might have a causal role in fibroblast–myofibroblast trans-differentiation in vitro. TGF-β treatment in primary human cardiac fibroblasts up-regulated the expression of several myofibroblast markers; VAP1 knockdown by siRNAs, however, markedly dampened the induction of myofibroblast markers by TGF-β (Fig. 2a). In addition, TGF-β-stimulated cell proliferation, as measured by EdU incorporation (Fig. 2b), cell migration (Fig. 2c), and cell contraction (Fig. 2d), were collectively weakened by VAP1 silencing.

Next, a novel mouse strain was created wherein exon 1 of the *Vap1* gene was floxed (Fig. 2e). Primary cardiac fibroblasts isolated from the Vap1^f/f^ mice were depleted of VAP1 by adenoviral Cre transduction; VAP1 ablation, as VAP1 knockdown in human cells, similarly down-regulated expression of myofibroblast markers (Fig. 2f) and retarded cell proliferation (Fig. 2g), cell migration (Fig. 2h), and cell contraction (Fig. 2i). Likewise, VAP1 deletion prevented gene expression and behavioral changes induced by Ang II (Supplementary Fig. 3) and ET-1 (Supplementary Fig. 4) in quiescent cardiac fibroblasts. By contrast, VAP1 over-expression accelerated fibroblast–myofibroblast transition in primary murine cardiac fibroblasts exposed to TGF-β (Fig. 2j–m). Notably, over-expression of a wild-type VAP1, but not an enzymatically inactive VAP1 (ref. ^30^) in MKL1-null fibroblasts, largely rescued the deficiency of myofibroblast maturation, suggesting that VAP1, as a downstream target of MKL1, may possibly mediate the profibrogenic effect of MKL1 in an enzymatic activity-dependent manner (Supplementary Fig. 5).

### Genetic manipulation of VAP1 in quiescent fibroblasts attenuates cardiac fibrosis in mice

**Fig. 3:**
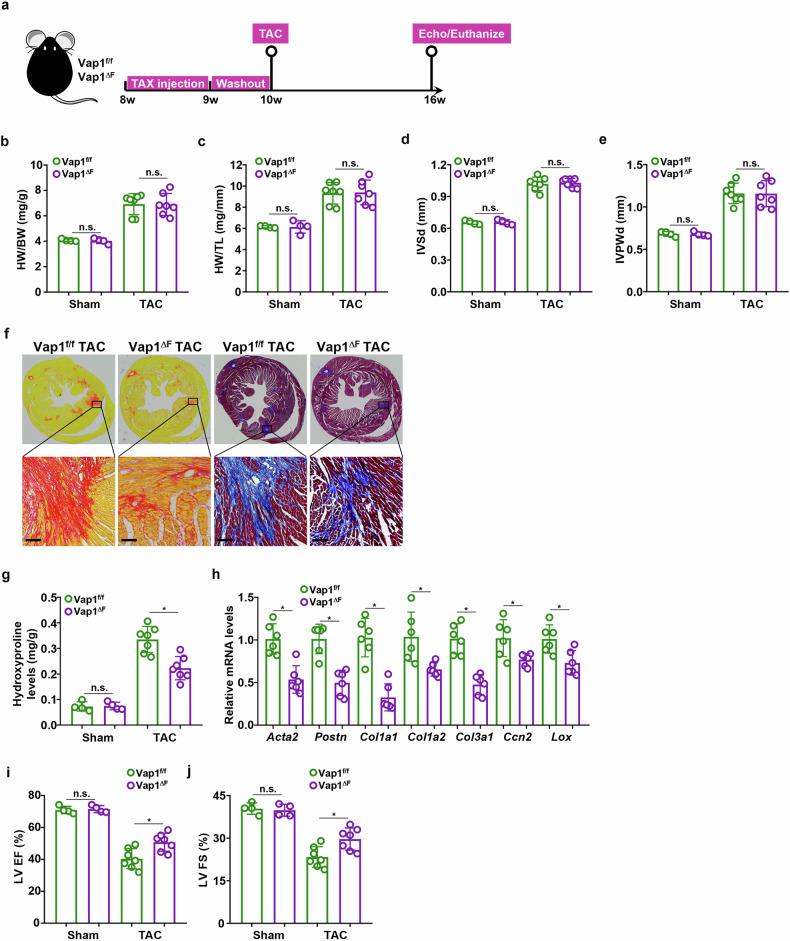
Genetic manipulation of VAP1 in quiescent fibroblasts attenuates cardiac fibrosis in mice*.* The Vap1^ΔF^ mice and the Vap1^f/f^ mice were subjected to the transverse aortic constriction (TAC) procedure to induce cardiac fibrosis and heart failure. Scheme of protocol (part **a**). Heart weight (HW) versus body weight (BW) ratio (part **b**). HW versus tibia length (TL) ratio (part **c**). Interventricular septal end diastole (IVSd) (part **d**). Interventricular posterior wall at end-diastolic dimension (IVPWd) (part **e**). Paraffin sections from the TAC-operated hearts were stained with Picrosirius Red or Masson’s trichrome (part **f**). Scale bar, 50 μm. Hydroxyproline levels (part **g**). Myofibroblast markers were examined by quantitative PCR (part **h**). Left ventricular ejection fraction (LVEF) (part **i**). Left ventricular fractional shortening (LVFS) (part **j**). *n* = 4 mice for the sham groups and *n* = 7 mice for the TAC groups. Scale bar, 50 μm. *P < 0.05, one-way analysis of variance with post hoc Scheffe’s test. NS not significant, VAP1 vascular adhesion protein 1.

To probe the contribution of VAP1 in quiescent fibroblasts to cardiac fibrosis, the Vap1^f/f^ mice were crossed to the *Col1a2*-Cre^ERT2^ mice to generate a fibroblast conditional VAP1 KO strain (Vap1^ΔF^). Because cardiomyopathy, including ischemic cardiomyopathy, dilated cardiomyopathy, hypertrophic cardiomyopathy, and restrictive cardiomyopathy, is considered a major cause for HF^31^, TAC procedure, frequently used to model non-ischemic cardiomyopathy^32–34^, was applied to both the Vap1^f/f^ mice and the Vap1^ΔF^ mice (Fig. 3a). Cardiac hypertrophy, as assessed by heart weight/body weight ratio (Fig. 3b), heart weight/tibia length ratio (Fig. 3c), interventricular septal end diastole (Fig. 3d), and interventricular posterior wall at end-diastolic dimension (Fig. 3e), was indistinguishable between the Vap1^ΔF^ mice and the Vap1^f/f^ mice post-surgery. PSR staining and Masson’s trichrome staining indicated that there was less extensive cardiac fibrosis in the Vap1^ΔF^ mice than in the Vap1^f/f^ mice (Fig. 3f). In addition, cardiac hydroxyproline quantification (Fig. 3g) and cardiac expression of myofibroblast markers (Fig. 3h) confirmed that VAP1 ablation in fibroblasts attenuated cardiac fibrosis. Importantly, amelioration of cardiac fibrosis in the Vap1^ΔF^ mice was accompanied by improved post-surgical heart function, as evaluated by left ventricular ejection fraction (LV EF) (Fig. 3i) and left ventricular fractional shortening (LV FS) (Fig. 3j), compared with the Vap1^f/f^ mice.

### Genetic manipulation of VAP1 in activated fibroblasts attenuates cardiac fibrosis in mice

**Fig. 4:**
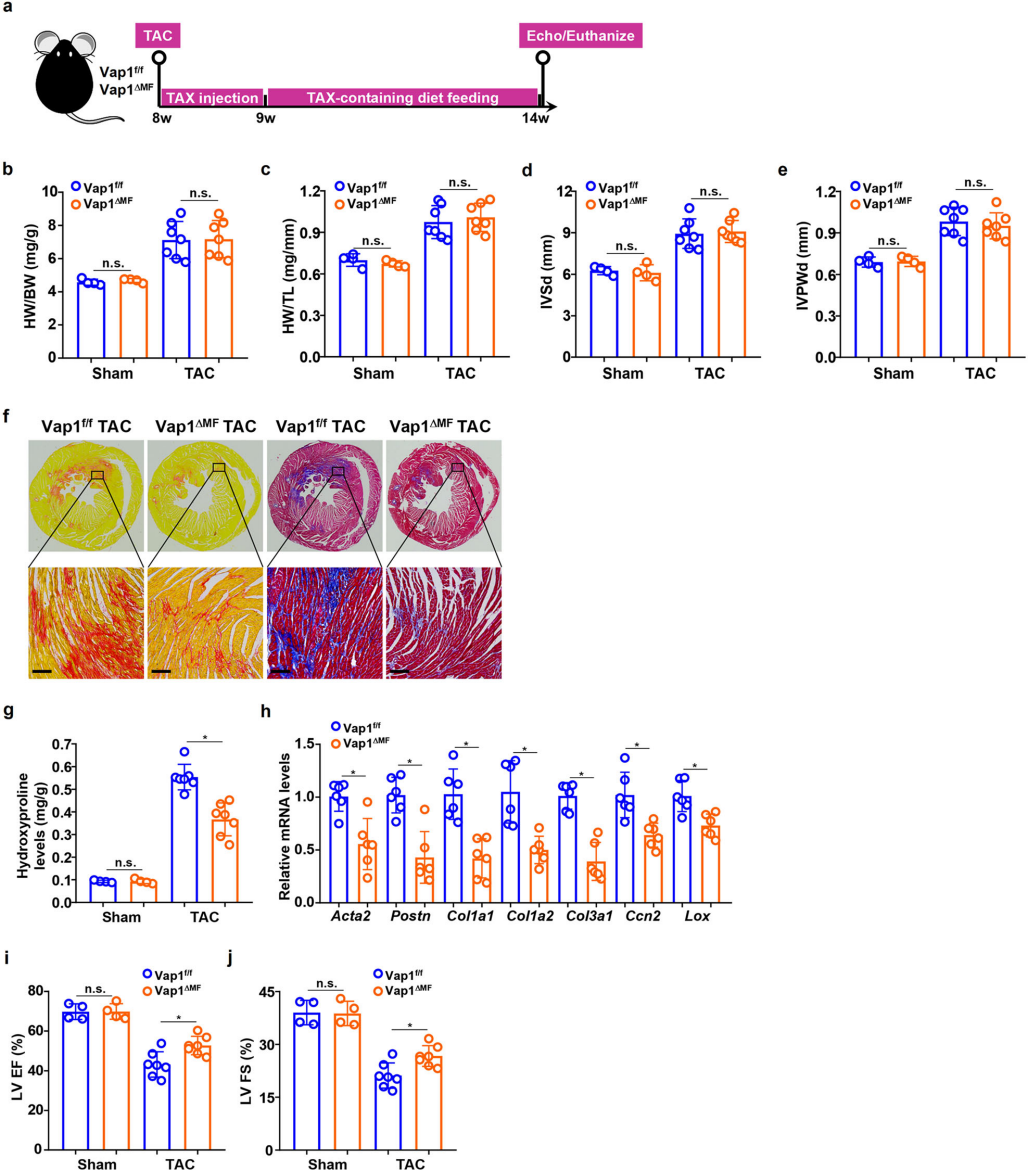
Genetic manipulation of VAP1 in activated fibroblasts attenuates cardiac fibrosis in mice. The Vap1^ΔMF^ mice and the Vap1^f/f^ mice were subjected to the transverse aortic constriction (TAC) procedure to induce cardiac fibrosis and heart failure. Scheme of protocol (part **a**). Heart weight (HW) versus body weight (BW) ratio (part **b**). HW versus tibia length (TL) ratio (part **c**). Interventricular septal end diastole (IVSd) (part **d**). Interventricular posterior wall at end-diastolic dimension (IVPWd) (part **e**). Paraffin sections from the TAC-operated hearts were stained with Picrosirius Red or Masson’s trichrome (part **f**). Scale bar, 50 μm. Hydroxyproline levels (part **g**). Myofibroblast markers were examined by quantitative PCR (part **h**). Left ventricular ejection fraction (LVEF) (part **i**). Left ventricular fractional shortening (LVFS) (part **j**). *n* = 4 mice for the sham groups and *n* = 7 mice for the TAC groups. Scale bar, 50 μm. *P < 0.05, one-way analysis of variance with post hoc Scheffe’s test. NS not significant, VAP1 vascular adhesion protein 1.

To probe the contribution of VAP1 in activated fibroblasts (myofibroblasts) to cardiac fibrosis, the Vap1^f/f^ mice were crossed to the *Postn*-Cre^ERT2^ mice to generate a myofibroblast conditional VAP1 KO strain (Vap1^ΔMF^); both the Vap1^f/f^ mice and the Vap1^ΔMF^ mice were subjected to the TAC procedure to induce cardiac fibrosis (Fig. 4a). It was observed that heart weight/body weight ratio (Fig. 4b), heart weight/tibia length ratio (Fig. 4c), and echocardiographic measurements of interventricular septal end diastole (Fig. 4d) and interventricular posterior wall at end-diastolic dimension (Fig. 4e) values were comparable between the Vap1^ΔΜF^ mice and the Vap1^f/f^ mice post-surgery, indicating that loss of VAP1 in myofibroblasts did not influence cardiac hypertrophy. PSR staining and Masson’s staining (Fig. 4f) combined with hydroxyproline quantification (Fig. 4g) detected fewer collagenous tissues in the Vap1^ΔΜF^ hearts than in the Vap1^f/f^ hearts. In addition, myofibroblast markers were down-regulated in the Vap1^ΔΜF^ hearts compared with the Vap1^f/f^ hearts (Fig. 4h). Better post-surgical heart functions, evidenced by LV EF values (Fig. 4i) and LV FS values (Fig. 4j), were recorded for the Vap1^ΔF^ mice than the Vap1^f/f^ mice.

### VAP1 regulates PDGFR signaling myofibroblasts

**Fig. 5:**
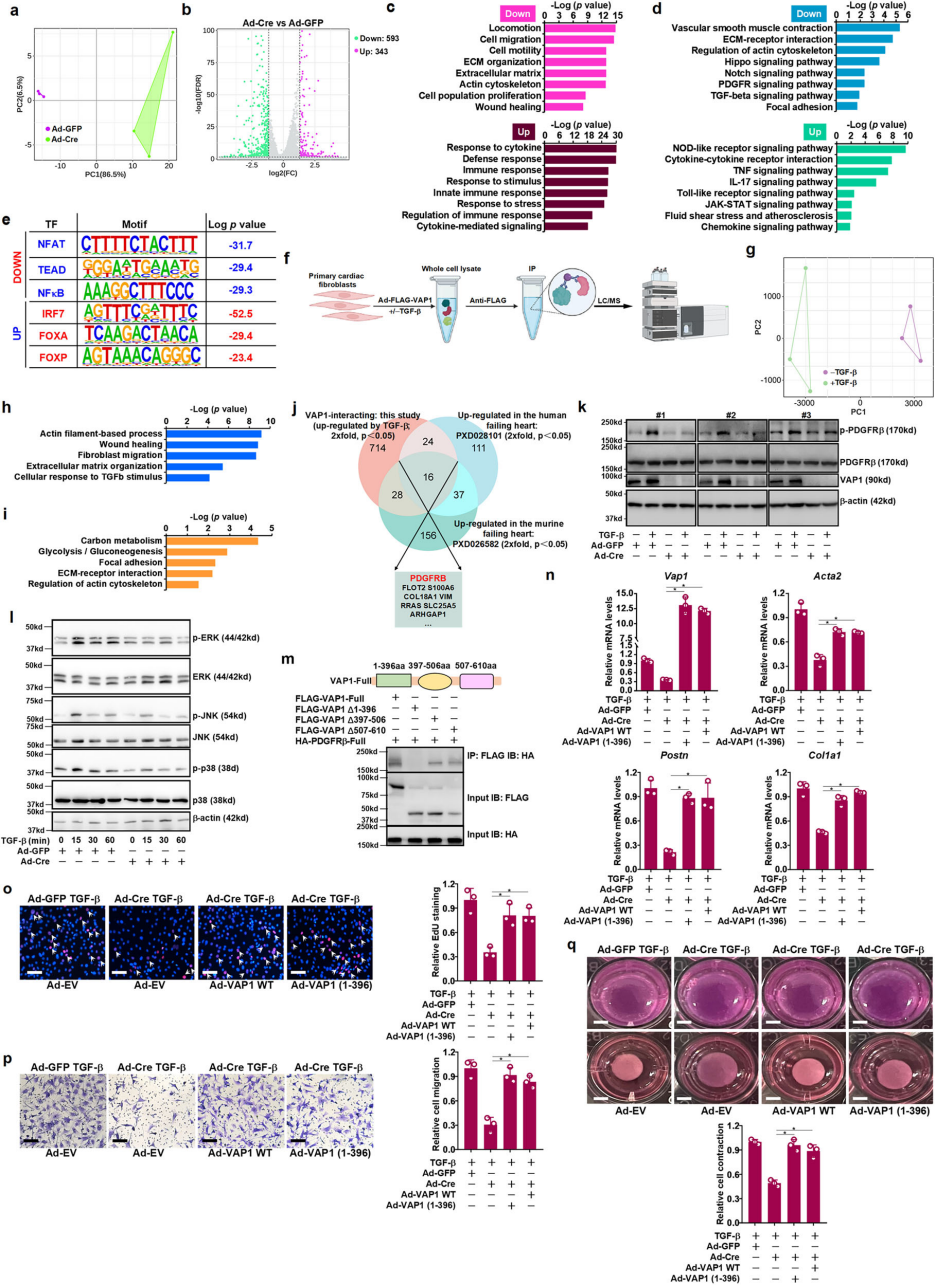
VAP1 regulates PDGFR signaling myofibroblasts*.* **a–e** Primary cardiac fibroblasts isolated from the Vap1^f/f^ mice were transduced with Ad-Cre or Ad-GFP followed by treatment with transforming growth factor-β (TGF-β) (5 ng/ml) for 24 h. RNA-sequencing was performed as described in Methods. Principal component analysis of transcriptome from VAP1-depleted cells (Ad-Cre) and control cells (Ad-GFP). *n* = 3 for each group (part **a**). Volcano plot showing differentially expressed genes in the VAP1-depleted cells versus the control cells (part **b**). Gene Ontology (GO, part **c**) and Kyoto Encyclopedia of Genes and Genomes (KEGG, part **d**) analyses of differentially expressed genes in the VAP1-depleted cells versus the control cells. HOMER analysis of differentially expressed genes in the VAP1-depleted cells versus the control cells (part **e**). **f–i** Primary murine cardiac fibroblasts were transduced with Ad-FLAG-VAP1 followed by treatment with or without TGF-β (5 ng/ml) for 24 h. Whole-cell lysates were immunoprecipitated with an anti-FLAG antibody and subjected to mass spectrometry as described in Methods. Schematic workflow (part **f**). Principal component analysis plot of VAP1-interacting proteins in the TGF-β-treated group (+TGF-β) and the control group (−TGF-β) (part **g**). GO analysis of differential VAP1-interacting proteins (part **h**). KEGG analysis of VAP1-interacting proteins (part **i**). **j** Venn diagram showing overlap between differential VAP1-interacting proteins and proteins upregulated in the myocardium per previously published proteomics data. **k**,**l** Primary cardiac fibroblasts isolated from the Vap1^f/f^ mice were transduced with Ad-Cre or Ad-GFP, followed by treatment with TGF-β (5 ng/ml) for 24 h. Protein phosphorylation was examined by western blotting. **m** Full-length platelet-derived growth factor receptor-β (PDGFRβ) vector and various VAP1 vectors were co-transfected into HEK293 cells. Immunoprecipitation was performed with anti-FLAG. **n**–**q** Primary cardiac fibroblasts isolated from the Vap1^f/f^ mice were transduced with Ad-Cre, Ad-GFP, Ad-VAP1 wild type (WT), or Ad-VAP1 (1–396) followed by treatment with TGF-β (5 ng/ml) for 24 h. Myofibroblast markers were examined by quantitative PCR (part **n**). Cell proliferation was examined by 5-ethynyl-2ʹ-deoxyuridine (EdU) incorporation. Scale bar, 50 μm (part **o**). Cell migration was examined by Boyden transwell assay. Scale bar, 50 μm (part **p**). Collagen contraction assay. Scale bar, 1 cm (part **q**). *n* = 3 biological replicates. Data are expressed as mean ± SD. *P < 0.05, one-way analysis of variance with post hoc Scheffe’s test. ECM extracellular matrix, EV, empty vector, FC fold change, FDR false discovery rate, IP immunoprecipitation, LC/MS liquid chromatography/mass spectrometry, NFAT nuclear factor of activated T cells, NF-κB nuclear factor kappa-B cells, TEAD TEA domain family members, TNF tumor necrosis factor, VAP1 vascular adhesion protein 1.

A multi-omics approach was exploited to pin down the mechanism whereby VAP1 might contribute to fibroblast activation. In the first arm of experiments, primary cardiac fibroblasts, with or without VAP1 deletion, were treated with TGF-β and then subjected to transcriptomic analysis by RNA-seq. VAP1 deletion profoundly altered cellular transcriptome, resulting in 936 genes being differentially regulated (Fig. 5a,b). GO analysis indicated that genes down-regulated by VAP1 deletion were primarily involved in cell migration, ECM synthesis, cytoskeletal remodeling, and cell proliferation, whereas genes up-regulated by VAP deletion were primarily involved in regulating the immune response (Fig. 5c). Consistently, KEGG analysis indicated that genes involved in well-established profibrogenic signaling pathways, including Notch signaling, TGF-β signaling, Hippo signaling, and PDGFR signaling, were down-regulated by VAP1 deletion, whereas genes contributing to the immune response, including tumor necrosis factor signaling, Toll-like receptor signaling, and JAK-STAT signaling, were up-regulated by VAP1 deletion (Fig. 5d). HOMER analysis indicated that top transcription factors whose activities were up-regulated by VAP1 deletion included nuclear factor of activated T cells (NFAT), TEA domain family members (TEAD), and nuclear factor kappa-B cells (NF-κB), whereas those whose activities were down-regulated by VAP1 deletion included interferon regulatory factor 7 (IRF7), forkhead transcription factor A (FOXA), and FOXP (Fig. 5e).

In the second arm of experiments, FLAG-VAP1 was virally transduced and over-expressed in cardiac fibroblasts followed by TGF-β treatment, immunoprecipitated by an anti-FLAG antibody, and then subjected to proteomic analysis by MS (Fig. 5f). TGF-β treatment significantly altered the VAP1 interactome (Fig. 5g). GO analysis indicated that these VAP1-interacting proteins altered by TGF-β treatment were primarily involved in cytoskeletal remodeling, wound healing, collagen production/deposition, fibroblast migration, and cellular response to growth factors (Fig. 5h). KEGG analysis indicated that the TGF-β-responsive VAP1 proteome might regulate cellular metabolism and ECM/cytoskeletal remodeling (Fig. 5i). We assumed that the VAP1-interacting protein that might modulate the profibrogenic activity of VAP1 should also be up-regulated during HF pathogenesis. Therefore, two previously published proteomics datasets were introduced for a combinatorial analysis: PXD028101 (https://www.iprox.cn/page/project.html?id=IPX0002777000) was derived from heart tissues of patients with HF caused by ischemic and non-ischemic dilative cardiomyopathy^35^, whereas PXD026582 (https://www.ebi.ac.uk/pride/archive/projects/PXD026582) was derived from heart tissues of mice induced to develop diabetic cardiomyopathy and HF^36^; 16 proteins, whose levels were higher in the failing hearts (identified by the two proteomics datasets) and which interacted with VAP1 more strongly in response to TGF-β treatment (detected by our own proteomics screening), were identified (Fig. 5j). We focussed on PDGFRβ because PDGFR signaling has a key role in tissue fibrosis and *PDGFRB*, which encodes PDGFRβ, can be used to label myofibroblast lineages^37,38^. Activation of the PDGFR signaling, as evidenced by PDGFRβ phosphorylation, was significantly weakened by Cre-mediated VAP1 deletion in cardiac fibroblasts (Fig. 5k). Additionally, MAPK activation as a downstream event of PDGFR signaling, including JNK phosphorylation, ERK phosphorylation, and p38 phosphorylation, was collectively inhibited by VAP1 deficiency (Fig. 5l). Fine mapping of the VAP1–PDGFRβ interaction by co-immunoprecipitation (Fig. 5m) revealed that VAP1 interacted with PDGFRβ via its N-terminal domain (1–396). Consistent with this observation, supplementation of a truncated VAP1 (1–396) was able to rescue the defects in fibroblast–myofibroblast transition of VAP1-depleted cells as potently as the wild-type VAP1 (Fig. 5n–q). Together, these data allude to a model wherein an interaction with VAP1 might enable PDGFRβ signaling to promote fibroblast activation.

### VAP1 inhibition attenuates cardiac fibrosis in mice

**Fig. 6:**
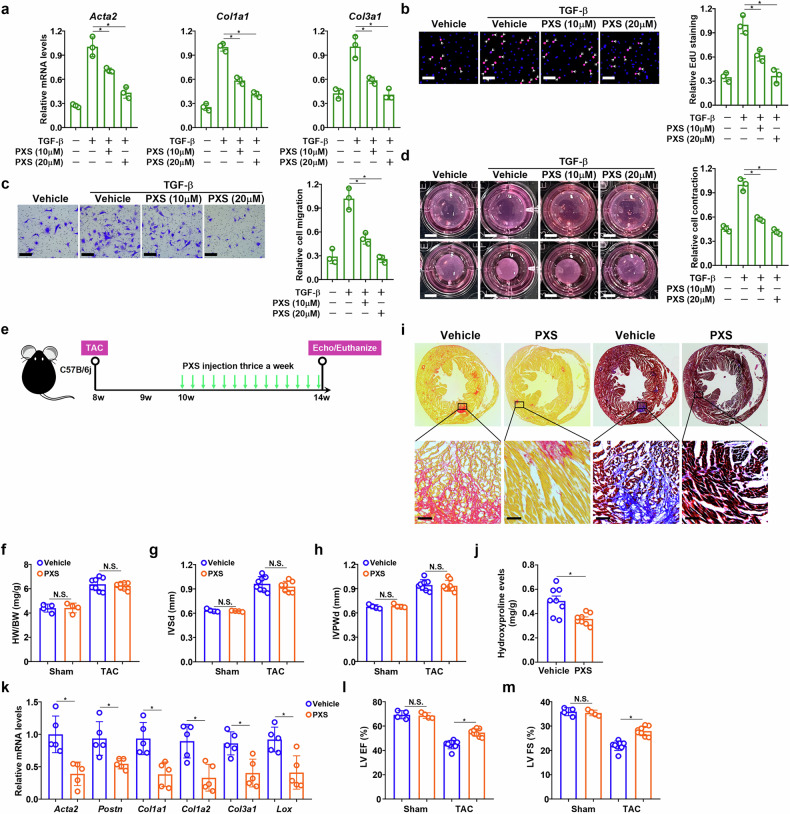
VAP1 inhibition attenuates cardiac fibrosis in mice. **a–d** Primary murine cardiac fibroblasts were treated with transforming growth factor-β (TGF-β) (5 ng/ml) with or without PXS for 24 h. Myofibroblast markers were examined by quantitative PCR (part **a**). Cell proliferation was examined by 5-Ethynyl-2ʹ-deoxyuridine (EdU) incorporation. Scale bar, 50 μm (part **b**). Cell migration was examined by Boyden transwell assay. Scale bar, 50 μm (part **c**). Collagen contraction assay. Scale bar, 1 cm (part **d**). n = 3 biological replicates. Data are expressed as mean ± SD. *P < 0.05, one-way analysis of variance (ANOVA) with post hoc Scheffe’s test. **e–m** C57B/6j mice were subjected to the transverse aortic constriction (TAC) procedure to induce heart failure followed by intervention with PXS. Scheme of protocol (part **e**). Heart weight (HW) versus body weight (BW) ratio (part **f**). Interventricular septum thickness (part **g**). Inferior posterior wall width in diastole (part **h**). Picrosirius Red staining and Masson’s staining. Scale bar, 50 μm (part **i**). Hydroxyproline levels (part **j**). Myofibroblast markers were examined by quantitative PCR (part **k**). Ejection fraction (part **l**). Fractional shortening (part **m**). *n* = 4–8 mice for each group. Data are expressed as mean ± SD. *P < 0.05, Student’s *t*-test. IVPWd interventricular posterior wall at end-diastolic dimension, IVSd interventricular septal end diastole, NS not significant VAP1 vascular adhesion protein 1.

To investigate the translational potential of targeting VAP1 for the intervention of adverse ventricular remodeling and HF, a small-molecule VAP1 inhibitor (PXS-4728A)^39^ was used. In cultured primary cardiac fibroblasts, PXS treatment dose-dependently repressed myofibroblast markers and retarded cell proliferation/migration/contraction in response to TGF-β treatment (Fig. 6a–d). Next, C57B/6j mice were subjected to the TAC procedure followed by PXS administration to evaluate whether VAP1 inhibition could suppress cardiac fibrosis and avert HF (Fig. 6e). Similar to VAP1 deletion in fibroblasts or myofibroblasts, VAP1 inhibition by PXS minimally influenced cardiac hypertrophy as assessed by heart weight/body weight ratio (Fig. 6f) and heart chamber expansion (Fig. 6g,h). On the contrary, histological staining (Fig. 6i), hydroxyproline quantification (Fig. 6j), and measurements of myofibroblast marker expression (Fig. 6k) all pointed to an attenuated phenotype of cardiac fibrosis. Consequently, the PXS-injected mice displayed better postsurgical heart functions, as measured by LV EF values (Fig. 6l) and LV FS values (Fig. 6m), than the vehicle-injected mice.

## Discussion

Fibroblast–myofibroblast transition is by now considered a paradigm in the pathogenesis of myocardial fibrosis and HF. In the present study, we detail a fibroblast/myofibroblast-autonomous regulatory role for VAP1 in this process. We first show that VAP1 is a direct transcription target of MKL1, a master regulator of tissue fibrosis^40^. This observation is consistent with what has been reported by Rippe et al. that VAP1 can be transcriptionally activated by MKL1 in vascular smooth muscles. Indeed, VAP1 over-expression partially overcomes the deficiency of myofibroblast maturation in MKL1-null fibroblasts, suggesting that MKL1 and VAP1 are likely interconnected functionally. It should be noted that the functional interplay between MKL1 and VAP1 may extend beyond fibroblasts/myofibroblasts. Both MKL1 and VAP1 have been noted in their role in mediating endothelium-dependent recruitment of myeloid cells from the circulation by regulating the expression of adhesion molecules^30,41^. Because pharmaceutical blockade of adhesion molecules (for example, ICAM1 (ref. ^42^) and VCAM1 (ref. ^43^)) are associated with amelioration of cardiac fibrosis and improvement of HF following myocardial injuries, it is reasonable to postulate that the MKL1–VAP1 axis may integrate multiple propathogenic processes to contribute to cardiac fibrosis and thus represent an attractive target for devising interventional solutions.

We show here that conditional deletion of VAP1 from quiescent fibroblasts or activated fibroblasts (myofibroblast) is equally effective in mitigating cardiac fibrosis. Recently, Yang and co-workers have reported that VAP1 knockdown attenuates post-infarct myocardial fibrosis in mice, thus essentially reaching the same conclusion as ours here^44^. However, because VAP1 knockdown in the study of Yang et al.^44^ was achieved by lentiviral delivery of short hairpin RNA under the control of a universal promoter, a system with notoriously nonspecific (off-target) effects, it is difficult to attribute the observed phenotype singularly to fibroblasts/myofibroblasts. Further compounding this issue is the observation by these authors that VAP1 knockdown simultaneously influenced cardiac hypertrophy and cardiac fibrosis, suggesting that amelioration of cardiac fibrosis might be secondary to (diminished) cardiac hypertrophy. Instead, our data show that fibroblast/myofibroblast-specific VAP1 deletion dampens cardiac fibrosis without altering cardiac hypertrophy. Therefore, even if VAP1 has a regulatory role in cardiomyocytes, a point that has yet to be conclusively proven, it can clearly be separated from a role in fibroblasts, which is sufficient to drive fibroblast–myofibroblast transition and consequently cardiac fibrosis. It might be worthwhile to continue this line of investigation, now that we have generated a never-before-reported Vap1^f/f^ strain for this study, to fully understand the lineage-specific contribution of VAP1 to cardiac fibrosis and HF.

One of the most noteworthy findings of this study is that VAP1 apparently can interact with PDGFRβ to potentiate PDGFRβ signaling. The underlying mechanism, however, is not clearly defined at this point. The observations that an enzymatically inactive form of VAP1 fails to compensate for MKL1 deletion and that a small-molecule VAP1 inhibitor could potently suppress fibroblast activation in vitro and cardiac fibrosis in vivo appear to imply that VAP1 might modulate PDGFRβ signaling in catalysis-dependent manner. Previously, it has been shown that VAP1 controls leukocyte rolling on the endothelium by oxidizing the amine group of amino acid residues (deamination); simulation analysis indicates that a lysine residue would most likely fit into the active site of VAP1 and thus serve as a de novo VAP1 substrate^14^. Of interest, a lysine residue (K567) is among the multiple disease-prone mutation sites that have been identified within the extracellular domain of PDGFRβ; mutation of this specific lysine residue seems to cause hyperactivation of PDGFRβ in patients with myofibroma^45^. Therefore, it is tempting to speculate that VAP1 might post-translationally modify PDGFRβ to modulate its activity. Alternatively, one of the end-products of VAP1-dependent catalysis is hydrogen peroxide (H_2_O_2_)^46^, a major source of reactive oxygen species. Reactive oxygen species can potentially activate PDGFR signaling either by promoting tyrosine phosphorylation of PDGFRβ^47^ or by oxidizing and thus deactivating the inhibitory phosphatase SHP-2 (ref. ^48^). In addition, H_2_O_2_ can independently contribute to fibroblast activation by promoting ECM synthesis and cell proliferation^49^. Thus, it is reasonable to propose that VAP1 might fine-tune PDGFRβ signaling by modulating cellular redox status. Of note, HOMER analysis shows that VAP1 deletion likely dampens the activities of several transcription factors including NFAT, TEAD, and NF-κB, which we contend is compatible with the VAP1–PDGFRβ theme. NFAT, known to directly modulate the fibroblast phenotype to contribute to cardiac fibrosis^50,51^, is primarily activated by Ca^2+^-dependent calcineurin-mediated de-phosphorylation^52^. Because PDGFRβ can signal through phospholipase C gamma to increase intracellular Ca^2+^ influx^53^, it is reasonable to postulate that NFAT might be downstream of the VAP1–PDGFRβ axis to mediate the pro-fibrogenic effects of VAP1. In the same vein, there is increasing evidence to suggest that elevated TEAD1 activity contributes to cardiac fibrosis^54,55^. Because PDGRFβ signaling can activate YAP, the primitive TEAD cofactor, to influence TEAD activity^52^, it is equally plausible that the VAP1–PDGFRβ axis may rely on TEAD to relay the pro-fibrogenic cues to the nucleus to program fibroblast–myofibroblast transition. These intriguing possibilities certainly deserve additional attention in future studies.

There are certain limitations of the present study that might dampen its translational potential. First, most of our conclusions rest upon a single animal model, which does not encapsulate the pathogenesis of cardiac fibrosis and HF in its entirety. Additional in vivo modeling is required to put our findings in better context. Second, although primary human cardiac fibroblasts were used in this study, the relevance of our findings to human disease is slim and should be validated in specimens taken from patients with HF. Third, the off-target effects of the supposedly “specific” VAP1 inhibitor used in this study cannot be conclusively excluded. Of note, two phase II clinical trials, aiming to test the efficacy of targeting VAP1 with either a small-molecule inhibitor^56^ or a monoclonal antibody^57^ for the intervention of liver fibrosis in the context of non-alcoholic steatohepatitis and primary sclerosing cholangitis, respectively, have recently been concluded with some promising but mixed results. Our data as summarized here certainly further fuel the enthusiasm for leveraging VAP1 inhibition against HF.

## Supplementary information

Supplementary Information

## Data Availability

The data that support the findings of this study are available upon reasonable request.
