# Supplementary material for: VAP1 promotes cardiac fibrosis by enabling PDGFR signaling in myofibroblasts
Source: Exp Mol Med. 2026 Apr 20;58(4):1284–96. doi: 10.1038/s12276-026-01690-7 (PMC13144515; doi:10.1038/s12276-026-01690-7)
Supplement: Supplementary file 1 — Supplementary Information [file 12276_2026_1690_MOESM1_ESM.pdf]

Huang S et al: VAP1 promotes cardiac fibrosis by enabling PDGFR signaling in myofibroblasts

Online supplementary material

Supplementary Figures: 5

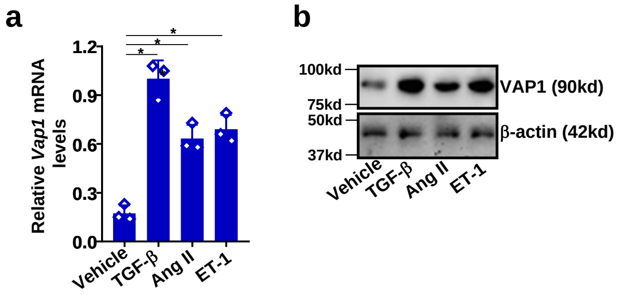

Supplementary Fig. 1: (**a, b**) Primary murine cardiac fibroblasts were treated with TGF-β (5ng/ml), Ang II (1μM), or ET-1 (1μM) for 24h. VAP1 expression was examined by qPCR (a) and Western blotting (b). N=3 biological replicates. Data are expressed as mean±S.D. *, $p < 0.05$, one-way ANOVA with *post-hoc* Scheffe´s test.

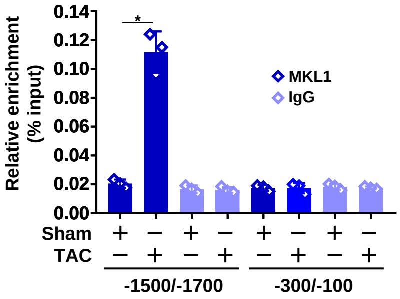

Supplementary Fig. 2: C57B/6j mice were subjected to the TAC procedure or the sham procedure. The mice were sacrificed 4 weeks after the surgery and ChIP assays were performed using heart tissues with anti-MKL1 or IgG. N=3 mice for each group. Data are expressed as mean±S.D. *, $p < 0.05$, one-way ANOVA with *post-hoc* Scheffe´s test.

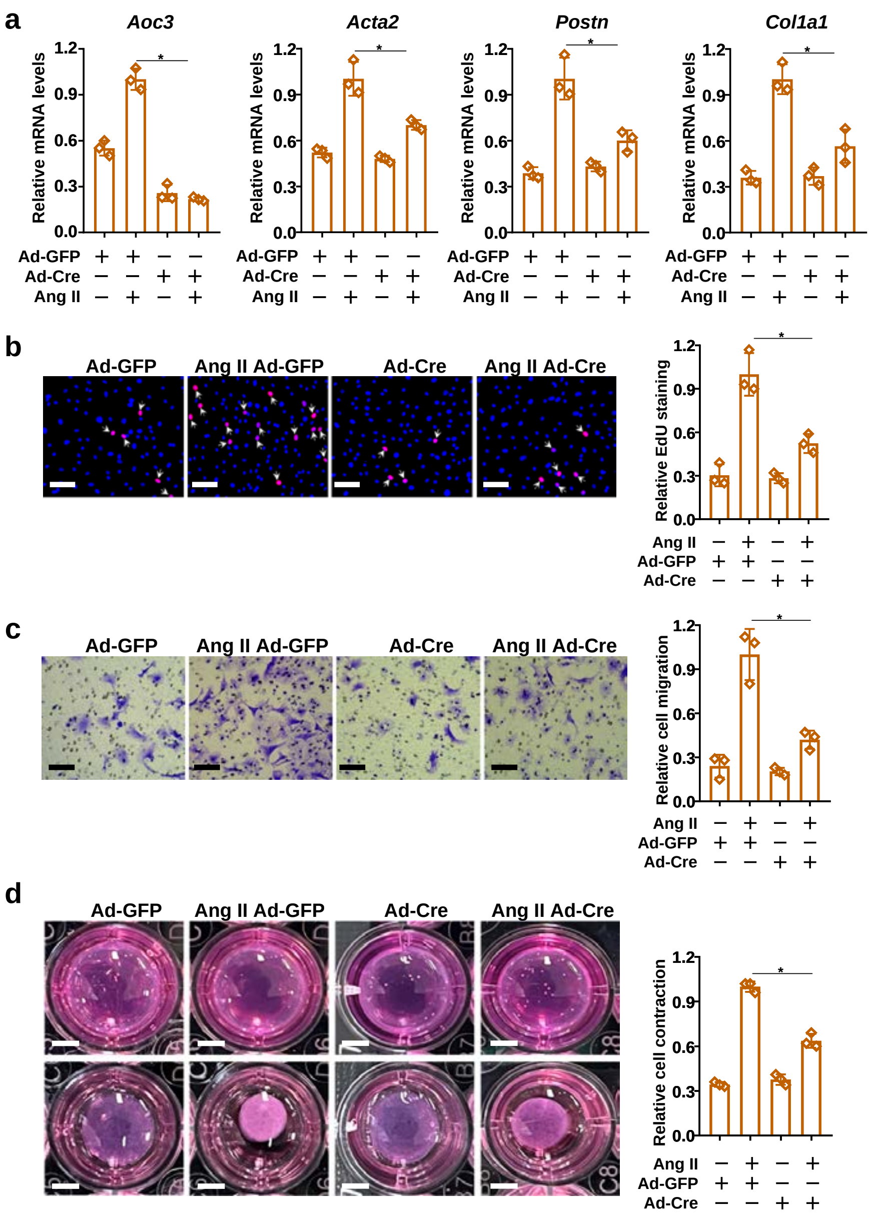

Supplementary Fig. 3: Primary cardiac fibroblasts isolated from the Vap1$^{f/f}$ mice were transduced with Ad-Cre or Ad-GFP followed by treatment with Ang II (1μM) for 24h. (**a**) Myofibroblast markers were examined by qPCR. (**b**) EdU incorporation assay. Scale bar, 50μm. (**c**) Transwell assay. Scale bar, 50μm. (**d**) Collagen contraction assay.Scale bar, 1cm. N=3 biological replicates. Data are expressed as mean±S.D. *, $p < 0.05$, one-way ANOVA with *post-hoc* Scheffe´s test.

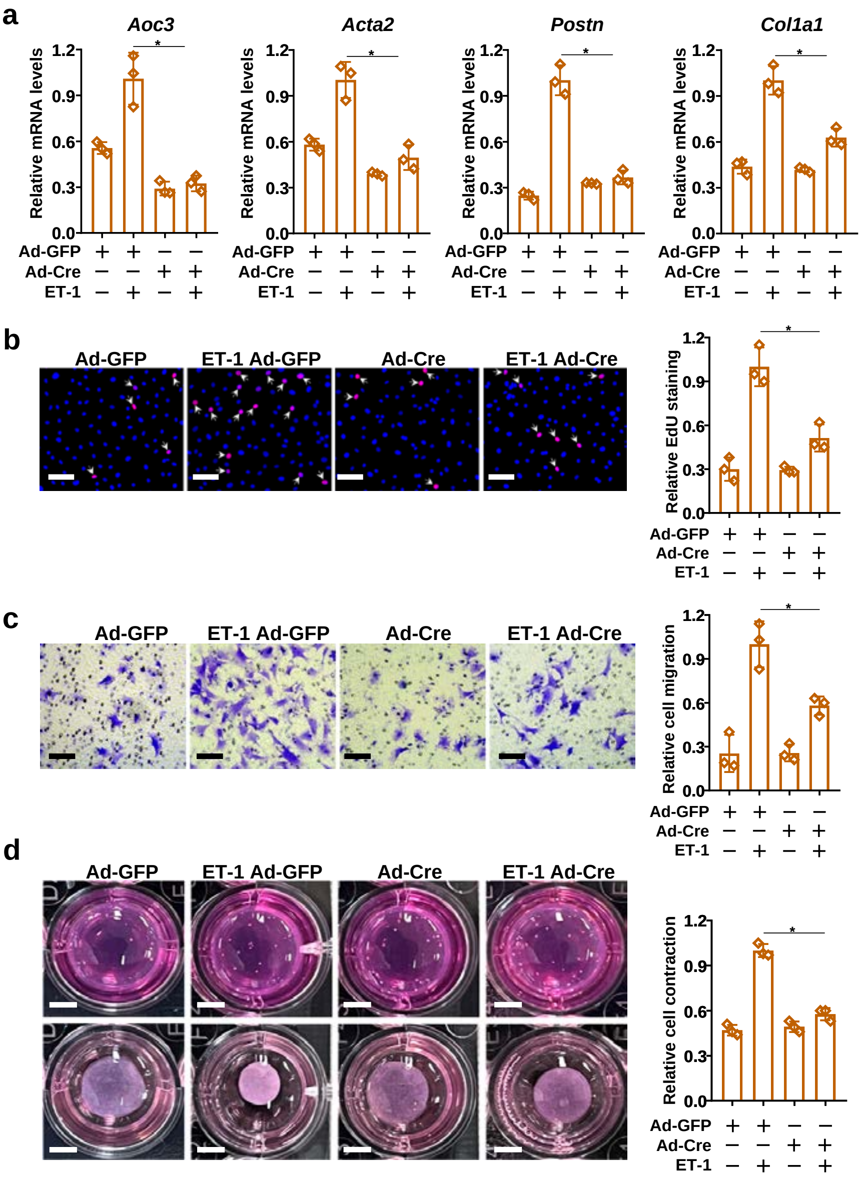

Supplementary Fig. 4: Primary cardiac fibroblasts isolated from the Vap1$^{f/f}$ mice were transduced with Ad-Cre or Ad-GFP followed by treatment with ET-1 (1μM) for 24h. (**a**) Myofibroblast markers were examined by qPCR. (**b**) EdU incorporation assay. Scale bar, 50μm. (**c**) Transwell assay. Scale bar, 50μm. (**d**) Collagen contraction assay.Scale bar, 1cm. N=3 biological replicates. Data are expressed as mean±S.D. *, $p<0.05$, one-way ANOVA with *post-hoc* Scheffe´s test.

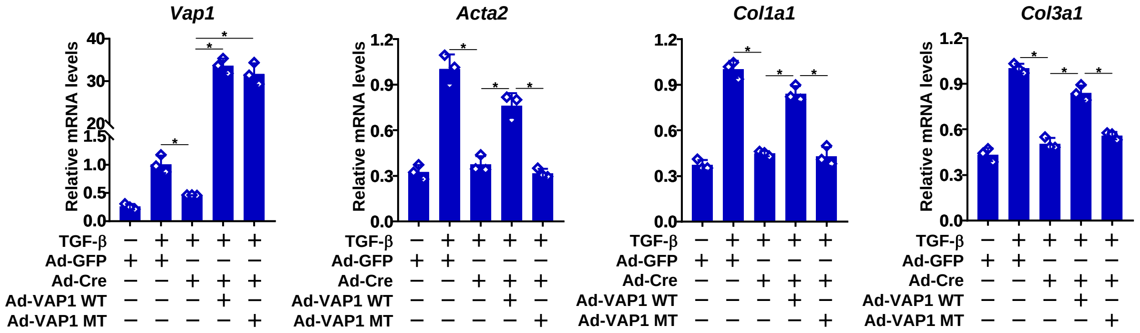

Supplementary Fig. 5: Primary cardiac fibroblasts isolated from $Mkl1^{f/f}$ mice were transduced with indicated adenovirus followed by treatment with TGF-β (5ng/ml) for 24h. Myofibroblast markers were examined by qPCR. N=3 biological replicates. Data are expressed as mean±S.D. *, $p < 0.05$, one-way ANOVA with *post-hoc* Scheffe´s test.

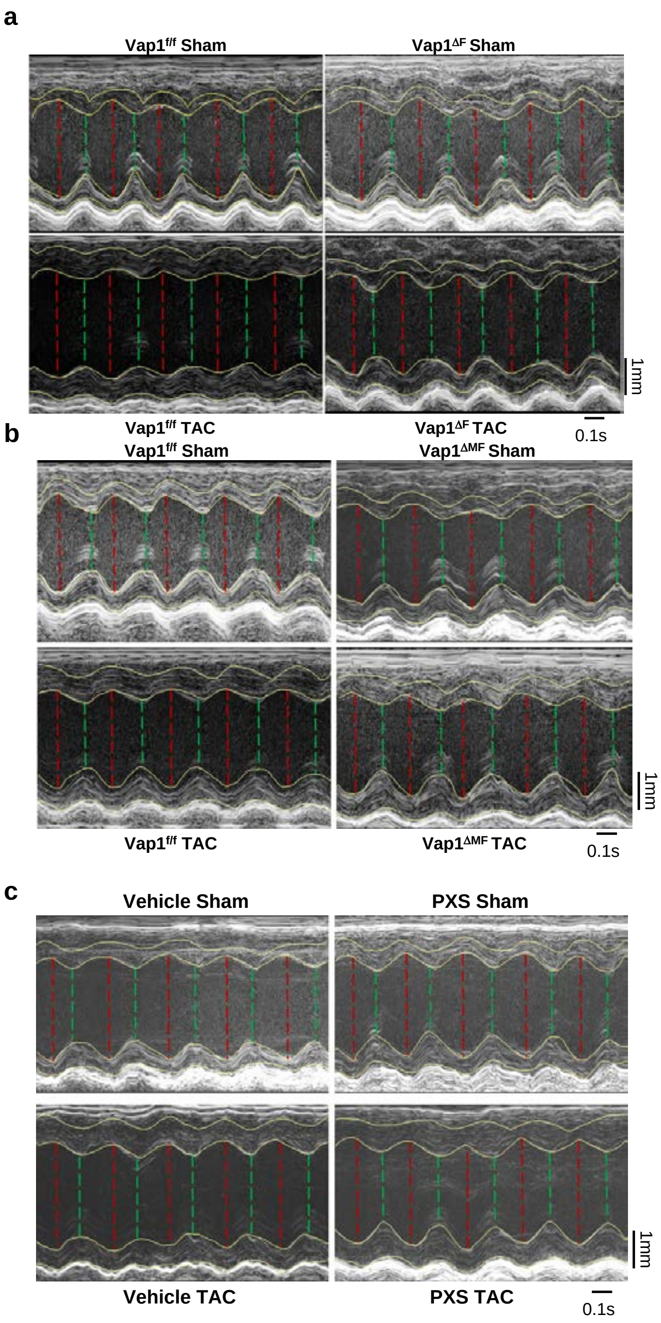

Supplementary Fig. 6: (**a**) Vap1$^{f/f}$mice andVap1$^{\Delta F}$mice were subjected to the TAC procedure to induce heart failure as described in Figure 3. Shown here are representative M-mode echocardiography images of the indicated groups. (**b**) Vap1$^{f/f}$mice andVap1$^{\Delta MF}$mice were subjected to the TAC procedure to induce heart failure as shown in Figure 4. Shown here are representative M-mode echocardiography images of the indicated groups. (**c**) C57B/6j mice were subjected to the TAC procedure to induce heart failure followed by intervention with PXS as shown in Figure 6. Shown here are representative M-mode echocardiography images of the indicated groups.
